# A new lanthanum(III) complex containing acetyl­acetone and 1*H*-imidazole

**DOI:** 10.1107/S205698901701461X

**Published:** 2017-10-20

**Authors:** Atsuya Koizumi, Takuya Hasegawa, Atsushi Itadani, Kenji Toda, Taoyun Zhu, Mineo Sato

**Affiliations:** a Graduate School of Science and Technology, Niigata University, 8050 Ikarashi 2-nocho, Niigata 950-2181, Japan; b Department of Marine Resource Science, Faculity of Agriculture and Marine Science, Kochi University, 200 Otsu, Monobe, Nankoku City, Kochi 783-8502, Japan; c Kochi University, 2-5-1 Akebono-cho, Kochi 780-8072, Japan; d Department of Human Sciences, Obihiro University of Agriculture and Veterinary Medicine, Inada-cho, Obihiro, Hokkaido 080-8555, Japan; e Nenjiang Senior High School, Nenjiang Heihe City, Heilongjiang Province 161400, People’s Republic of China; f Department of Chemistry and Chemical Engineering, Faculty of Engineering, Niigata University, Ikarashi 2-no-cho, Niigata City 950-2181, Japan

**Keywords:** crystal structure, lanthanum complex, acetyl­acetone, imidazole

## Abstract

In the title complex, di­aqua­(1*H*-imidazole-κ*N* ^3^)(nitrato-κ^2^ *O*,*O*′)bis­(4-oxo­pent-2-en-2-olato-κ^2^ *O*,*O*′)lanthanum(III), [La(C_5_H_7_O_2_)_2_(NO_3_)(C_3_H_4_N_2_)(H_2_O)_2_], the La atom is coordinated by eight O atoms of two acetyl­acetonate (acac) anions acting as bidentate ligands, two water mol­ecule as monodentate ligands, one nitrate anions as a bidentate ligand and one N atom of an imidazolate (ImH) molecule as a monodentate ligand. Thus, the coordination number of the La atom is nine in a monocapped square anti­prismatic polyhedron. There are three types of inter­molecular hydrogen bonds between ligands, the first involving nitrate–water O⋯H—O inter­actions running along the [001] direction, the second involving acac–water O⋯H—O inter­actions along the [010] direction and the third involving an Im–nitrate N—H⋯O inter­action along the [100] direction (five inter­actions of this type). Thus, an overall one-dimensional network structure is generated. The mol­ecular plane of an ImH molecule is almost parallel to that of a nitrate ligand, making an angle of only 6.04 (12)°. Inter­estingly, the ImH plane is nearly perpendicular to the planes of two neighbouring acac ligands.

## Abstract 2

The title complex is coordinated by two acetyl­acetonate, one 1*H*-imidazole, one nitrate and one water ligand. The mol­ecular plane of the imidazole ligand is almost parallel to that of the nitrate anion.

## Chemical context   

Carb­oxy­lic acid-based linkers are often used in metal–organic complexes involving rare earth elements because they can easily build a framework structure due to the oxophilic nature of lanthanide ions. Recently, some imidazole-based metal organic complexes were reported to form such framework structures (Zurawski *et al.*, 2011 ▸). A remarkable feature of imidazole-based compounds is the ability to form porous networks, such as zeolitic imidazolate frameworks (ZIFs) (Zurawski *et al.*, 2012 ▸; Müller-Buschbaum *et al.*, 2015 ▸), which show a good performance for gas adsorption with feasible chemical and thermal stability. For example, ZIF-8 and ZIF-11 have a remarkable chemical resistance to boiling alkaline water and organic solvents, and high thermal stability up to 823 K (Park *et al.*, 2006 ▸; Zhong *et al.*, 2014 ▸). Another inter­esting feature of these complexes is that they exhibit luminescence based on *f*–*f* transitions of lanthanides assisted by the ligand antenna effect (Rybak *et al.*, 2012 ▸). The complexes of rare earth atoms with β-diketonates have been investigated widely because of their simple use as organic ligands (Binnemans, 2005 ▸). These ligands can give an increase in luminescence efficiency and intensity, Eu(acac)_3_ (acac is acetyl­acetonate) being one such complex (Kuz’mina & Eliseeva, 2006 ▸). In addition, Tb(acac)_3_ is used as an active light-emitting layer in the first LED based on lanthanide complexes (Kido *et al.*, 1990 ▸). From the viewpoint of high luminescence efficiency, the luminescence based on the *f*–*d* transition of Ce^3+^ is quite promising due to its allowed electronic transition. However, the emission of Ce^3+^ in metal–organic complexes have been reported only occasionally, for example, in [Ce(triRNTB)_2_](CF_3_SO_3_)_3_ [NTB = *N*-substituted tris­(*N*-alkyl­benzimidazol-2-ylmeth­yl)amine] and _∞_^3^[Ce(Im)_3_(ImH)]·ImH (Zheng *et al.*, 2007 ▸; Meyer *et al.*, 2015 ▸). One of the reasons for this is the difficulty of retaining a certain distance between Ce^3+^ ions in order to avoid luminescence quenching caused by energy transfer between Ce^3+^ ions. [Ce(triRNTB)_2_](CF_3_SO_3_)_3_ shows a blue emission accompanied by neighbouring Ce⋯Ce distance of about 17∼18 Å. NTB is a bulky ligand so that it can keep the neighbouring central ions far away. Also, it may be important for the emission of Ce^3+^ to construct a structure of isolated entities rather than a framework structure, which does not necessarily guarantee a sufficient long metal–metal distance. During the investigation of the synthesis of lanthanide complexes for Ce^3+^ emission using functional ligands, like imidazole with the antenna effect, as well as β-diketone derivatives, we have synthesized a novel lanthanum complex, although the cerium derivative has not been synthesized yet. This study reports structural data on a newly synthesized lanthanum complex comprising functional ligands of imidazole and acetyl­acetone.
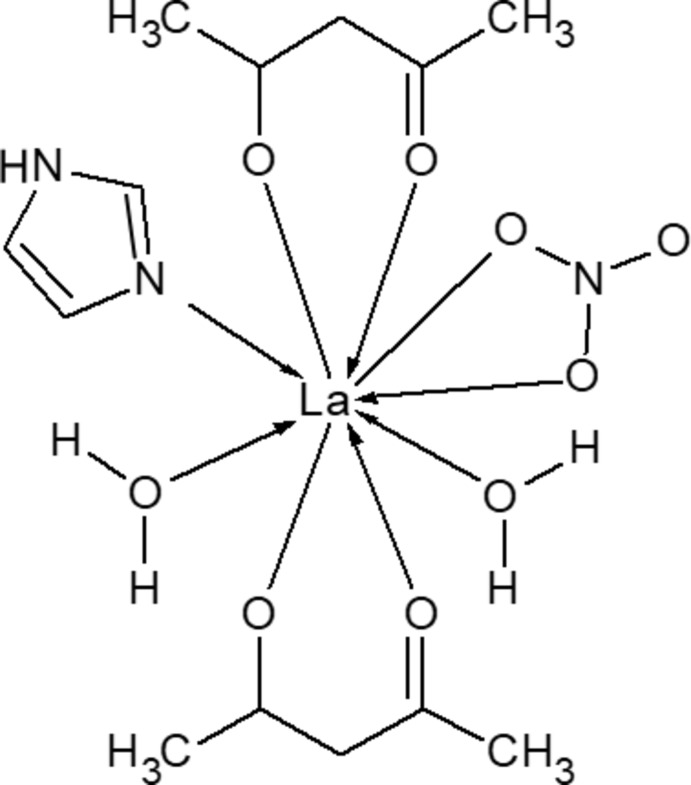


## Structural commentary   

**Figure 1:**
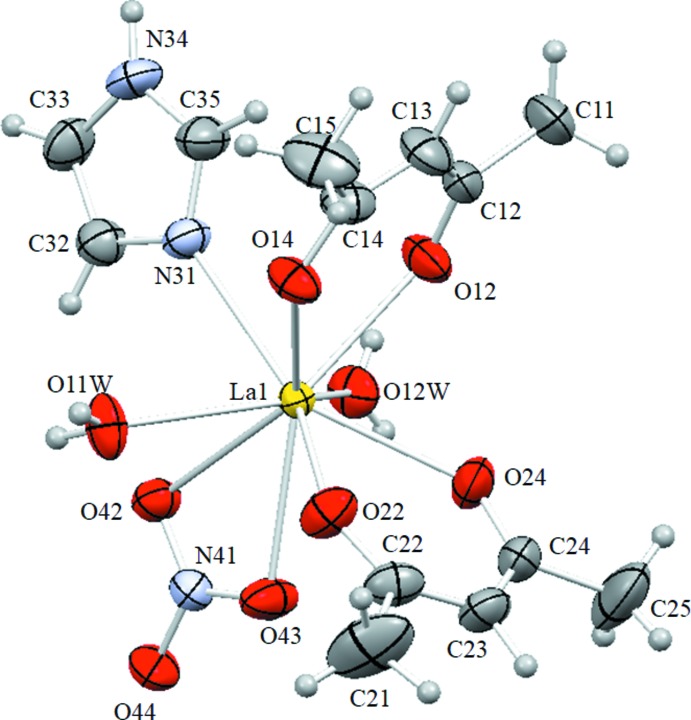
View of the mol­ecular structure of the title complex, with displacement ellipsoids for non-H atoms drawn at the 50% probability level.

The title complex crystallizes in the monoclinic space group *P*2_1_/*c*, with one formula unit of [La(C_5_H_7_O_2_)_2_(NO_3_)(C_3_H_4_N_2_)(H_2_O)_2_]. Each mol­ecule is isolated individually, *i.e.* the structure is not a framework structure. The central La atom is coordinated by eight O atoms from two acac anions, two water mol­ecules, one nitrate anion and one N atom from one Im ligand (Fig. 1 ▸). Thus, the La atom has a monocapped square anti­prismatic coordination. The La—O bond lengths can be classified into three categories; the first concerns inter­actions with a bidentate acac mol­ecule, the second those with a nitrate ion behaving as a bidentate ligand and the third those with a water mol­ecule. All the distances are quite comparable with the corresponding distances reported for acac complexes (Phillips *et al.*, 1968 ▸; Antsyshkina *et al.*, 1997 ▸; Fukuda *et al.*, 2002 ▸) and for nitrate complexes (Al-Karaghouli & Wood, 1972 ▸; Frechette *et al.*, 1992 ▸; Fukuda *et al.*, 2002 ▸). An Im ligand coordinates to the central La atom as a monodentate ligand. The La—N distance is comparable with that of _∞_^3^[Ce(Im)_3_(ImH)]·ImH (Meyer *et al.*, 2015 ▸).

## Supra­molecular features   

**Figure 2:**
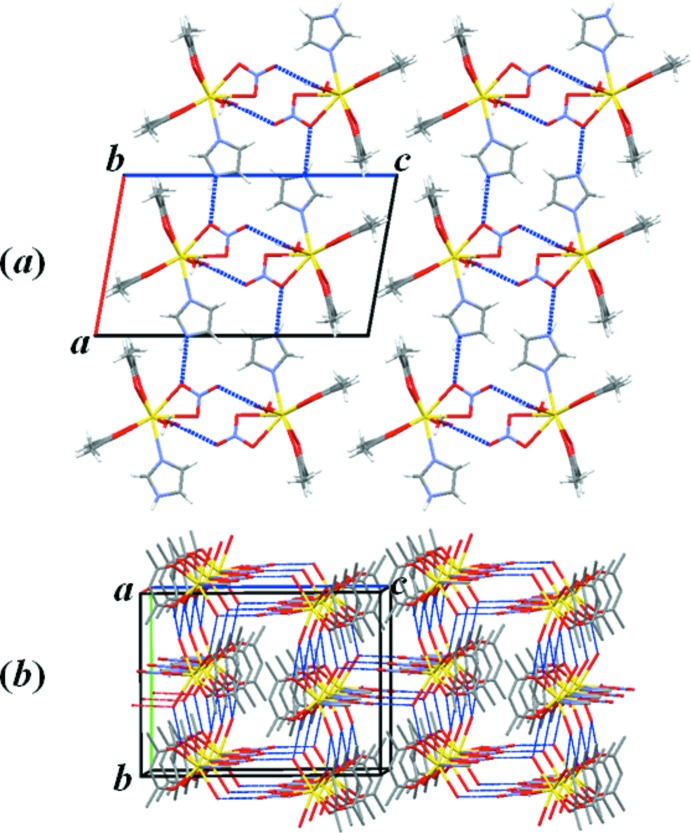
Connection of discrete complexes by inter­molecular hydrogen-bonding (blue dashed lines) chains in the *ac* plane projected (*a*) along the *b* axis and (*b*) along the *a* axis. Colour code: La yellow, C grey, N purple and O red. H atoms have been omitted.

**Figure 3:**
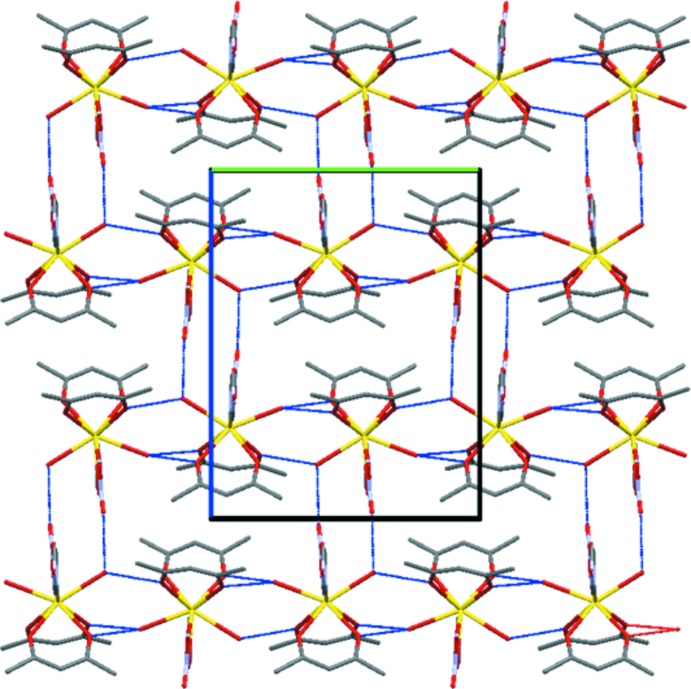
Connection of discrete complexes by inter­molecular hydrogen-bonding (blue dashed lines) chains in the *bc* plane. Colour code: La yellow, C grey, N purple and O red. H atoms have been omitted.

The discrete complexes are linked by five kinds of hydrogen bonds (Table 1 ▸). There are two types of hydrogen bond chains that lie nearly within the *ac* plane; the first type are the chains parallel to [100] by centrosymmetric pairs of inter­molecular O⋯H—N hydrogen bonds between the O atom of a nitrate anion and the H atom of an ImH ligand, and the other type are the chains parallel to [001], formed also by centrosymmetric pairs of inter­molecular O⋯H—O hydrogen bonds between the O atom of a nitrate anion and the H atom of a water mol­ecule (O12*W*) (Fig. 2 ▸ *a*). It is notable, as shown in Fig. 2 ▸(*b*), that these hydrogen bonds are both almost parallel to the *ac* plane. This arises from the fact that the angle difference between the mol­ecular planes of the nitrate and ImH mol­ecules is only 6.04 (12)°. Along the [010] direction, there are three types of hydrogen-bond chains, all of which are the hydrogen bond between the O atom of the acac anion and the H atom of water mol­ecule (Fig. 3 ▸). All the ligands coordinating to the central La atom are involved in hydrogen bonding with neighbouring complexes. In this way, all mol­ecules are connected by hydrogen bonds running in every axis direction, leading to a three-dimensional supra­molecular network structure. Furthermore, it should be mentionned that the mol­ecular plane of each ImH ligand is almost perpendic­ular to the mol­ecular planes of the two neighbouring acac anions, making angles of 84.68 (11) and 85.27 (11)°, respectively.

## Database survey   

The crystal structures of other related acac complexes including lanthanide ions have been reported (Berg & Acosta, 1968 ▸; Binnemans, 2005 ▸; Filotti *et al.*, 1996 ▸; Fujinaga *et al.*, 1981 ▸; Lim *et al.*, 1996 ▸; Phillips *et al.*, 1968 ▸; Richardson *et al.*, 1968 ▸; Stites *et al.*, 1948 ▸). The crystal structures of other related ImH complexes including lanthanide ions have also been reported (Dan *et al.*, 2004 ▸; Dechnik *et al.*, 2016 ▸; Meyer *et al.*, 2015 ▸; Pan *et al.*, 2016 ▸; Zhou *et al.*, 2008 ▸; Zurawski *et al.*, 2013 ▸).

## Synthesis and crystallization   

Colourless plate-like crystals were obtained by slow evaporation from a methanol solution of La(NO_3_)_3_·6H_2_O, acetyl­acetone and 1*H*-imidazole (1:5:5 molar ratio). The products were filtered off and dried at room temperature.

## Refinement   

Crystal data, data collection and structure refinement details are summarized in Table 2 ▸. H atoms bonded to C atoms were positioned geometrically after each cycle in idealized locations and refined as riding on their parent C atoms, with C—H = 0.93 Å and *U*_iso_(H) = 1.2*U*_eq_(C). H atoms bonded to water O atoms were located in a difference Fourier map, and isotropically refined without any distance restraint and with restraints of *U*_iso_(H) = 1.5*U*_eq_(O).

## Supplementary Material

Crystal structure: contains datablock(s) global, I. DOI: 10.1107/S205698901701461X/vn2131sup1.cif

Structure factors: contains datablock(s) I. DOI: 10.1107/S205698901701461X/vn2131Isup2.hkl

CCDC reference: 1579078

Additional supporting information:  crystallographic information; 3D view; checkCIF report

## Figures and Tables

**Table 1:** Hydrogen-bond geometry (Å, °)

*D*—H⋯*A*	*D*—H	H⋯*A*	*D*⋯*A*	*D*—H⋯*A*
N34—H34⋯O43^i^	0.86	2.15	2.942 (2)	153
O11*W*—H11*X*⋯O24^ii^	0.81 (3)	2.10 (3)	2.8353 (19)	152 (2)
O11*W*—H11*Y*⋯O12^ii^	0.81 (3)	2.00 (3)	2.8014 (19)	168 (3)
O12*W*—H12*Y*⋯O44^iii^	0.85 (3)	2.10 (3)	2.930 (2)	167 (3)
O12*W*—H12*X*⋯O22^iv^	0.73 (3)	2.30 (3)	3.0025 (19)	161 (3)

Symmetry codes: (i) 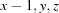; (ii) 
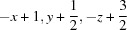
; (iii) 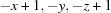; (iv) 
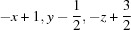
.

**Table 2:** Experimental details

Crystal data	
Chemical formula	[La(C_5_H_7_O_2_)_2_(NO_3_)(C_3_H_4_N_2_)(H_2_O)_2_]
*M*_r_	503.24
Crystal system, space group	Monoclinic, *P*2_1_/*c*
Temperature (K)	293
*a*, *b*, *c* (Å)	9.8233 (9), 12.4719 (12), 16.4432 (16)
β (°)	100.184 (7)
*V* (Å^3^)	1982.8 (3)
*Z*	4
Radiation type	Mo *K*α
μ (mm^−1^)	2.20
Crystal size (mm)	0.42 × 0.39 × 0.12

Data collection	
Diffractometer	XTALAB-MINI
Absorption correction	Multi-scan (*REQAB*; Rigaku, 1998 ▸)
*T*_min_, *T*_max_	0.456, 0.772
No. of measured, independent and observed [*I* > 2σ(*I*)] reflections	19723, 4543, 4317
*R*_int_	0.019
(sin θ/λ)_max_ (Å^−1^)	0.649

Refinement	
*R*[*F* ^2^ > 2σ(*F* ^2^)], *wR*(*F* ^2^), *S*	0.016, 0.040, 1.07
No. of reflections	4543
No. of parameters	251
H-atom treatment	H atoms treated by a mixture of independent and constrained refinement
Δρ_max_, Δρ_min_ (e Å^−3^)	0.51, −0.54

Computer programs: *CrystalClear* (Rigaku/MSC, 2006 ▸), *SORTAV* (Blessing, 1995 ▸), *SHELXS2013* (Sheldrick, 2008 ▸), *SHELXL2014* (Sheldrick, 2015 ▸) and *ORTEP-3 for Windows* and *WinGX* (Farrugia, 2012 ▸).

## supplementary crystallographic information

### Crystal data

**Table:**

[La(C_5_H_7_O_2_)_2_(NO_3_)(C_3_H_4_N_2_)(H_2_O)_2_]	*F*(000) = 1000
*M**_r_* = 503.24	*D*_x_ = 1.686 Mg m^−^^3^
Monoclinic, *P*2_1_/*c*	Mo *K*α radiation, λ = 0.71073 Å
Hall symbol: -P 2ybc	Cell parameters from 19036 reflections
*a* = 9.8233 (9) Å	θ = 3–27.5°
*b* = 12.4719 (12) Å	µ = 2.20 mm^−^^1^
*c* = 16.4432 (16) Å	*T* = 293 K
β = 100.184 (7)°	Prism, colorless
*V* = 1982.8 (3) Å^3^	0.42 × 0.39 × 0.12 mm
*Z* = 4	

### Data collection

**Table:**

XTALAB-MINI diffractometer	4543 independent reflections
Radiation source: sealed x-ray tube	4317 reflections with *I* > 2σ(*I*)
Graphite monochromator	*R*_int_ = 0.019
Detector resolution: 10 pixels mm^-1^	θ_max_ = 27.5°, θ_min_ = 3.0°
phi or ω oscillation scans	*h* = −12→12
Absorption correction: multi-scan (*REQAB*; Rigaku, 1998)	*k* = −16→16
*T*_min_ = 0.456, *T*_max_ = 0.772	*l* = −21→21
19723 measured reflections	

### Refinement

**Table:**

Refinement on *F*^2^	Primary atom site location: structure-invariant direct methods
Least-squares matrix: full	Secondary atom site location: difference Fourier map
*R*[*F*^2^ > 2σ(*F*^2^)] = 0.016	Hydrogen site location: mixed
*wR*(*F*^2^) = 0.040	H atoms treated by a mixture of independent and constrained refinement
*S* = 1.07	*w* = 1/[σ^2^(*F*_o_^2^) + (0.0179*P*)^2^ + 0.8453*P*] where *P* = (*F*_o_^2^ + 2*F*_c_^2^)/3
4543 reflections	(Δ/σ)_max_ < 0.001
251 parameters	Δρ_max_ = 0.51 e Å^−^^3^
0 restraints	Δρ_min_ = −0.54 e Å^−^^3^
0 constraints	

### Special details

**Table:**

Geometry. All esds (except the esd in the dihedral angle between two l.s. planes) are estimated using the full covariance matrix. The cell esds are taken into account individually in the estimation of esds in distances, angles and torsion angles; correlations between esds in cell parameters are only used when they are defined by crystal symmetry. An approximate (isotropic) treatment of cell esds is used for estimating esds involving l.s. planes.

### Fractional atomic coordinates and isotropic or equivalent isotropic displacement parameters (Å^2^)

**Table:**

	*x*	*y*	*z*	*U*_iso_*/*U*_eq_	
La1	0.49302 (2)	0.06987 (2)	0.73872 (2)	0.02341 (4)	
C11	0.3206 (3)	−0.1406 (2)	0.94575 (16)	0.0675 (7)	
H11A	0.2657	−0.1885	0.9076	0.101*	
H11B	0.272	−0.1238	0.9899	0.101*	
H11C	0.4071	−0.1742	0.9679	0.101*	
C12	0.3473 (2)	−0.03887 (17)	0.90146 (12)	0.0416 (4)	
O12	0.40186 (14)	−0.05003 (10)	0.83749 (8)	0.0404 (3)	
C13	0.3101 (2)	0.05849 (17)	0.93181 (13)	0.0489 (5)	
H13	0.2668	0.0566	0.9777	0.059*	
C14	0.33217 (18)	0.15900 (16)	0.89926 (11)	0.0395 (4)	
O14	0.38770 (15)	0.17374 (10)	0.83705 (8)	0.0454 (3)	
C15	0.2866 (2)	0.2580 (2)	0.93995 (16)	0.0622 (6)	
H15A	0.2468	0.3083	0.8983	0.093*	
H15B	0.3651	0.29	0.9745	0.093*	
H15C	0.2191	0.2386	0.973	0.093*	
C21	0.8806 (3)	0.2748 (2)	0.8716 (2)	0.0910 (11)	
H21A	0.8263	0.3193	0.9011	0.136*	
H21B	0.8983	0.3122	0.8235	0.136*	
H21C	0.9668	0.2578	0.9067	0.136*	
C22	0.80297 (19)	0.17260 (16)	0.84555 (12)	0.0424 (4)	
O22	0.67656 (13)	0.18282 (10)	0.81634 (9)	0.0448 (3)	
C23	0.8740 (2)	0.07563 (17)	0.85616 (14)	0.0456 (5)	
H23	0.9679	0.0784	0.878	0.055*	
C24	0.81648 (19)	−0.02525 (16)	0.83686 (12)	0.0425 (4)	
O24	0.69227 (13)	−0.04257 (11)	0.80450 (9)	0.0446 (3)	
C25	0.9079 (3)	−0.1227 (2)	0.8548 (2)	0.0856 (10)	
H25A	0.8662	−0.1726	0.8875	0.128*	
H25B	0.9968	−0.1014	0.8847	0.128*	
H25C	0.919	−0.1561	0.8038	0.128*	
N31	0.22279 (15)	0.07647 (12)	0.67424 (10)	0.0367 (3)	
C32	0.1625 (2)	0.07834 (15)	0.59247 (12)	0.0411 (4)	
H32	0.2101	0.0768	0.5484	0.049*	
C33	0.0238 (2)	0.08279 (17)	0.58553 (14)	0.0481 (5)	
H33	−0.0409	0.0853	0.5369	0.058*	
N34	−0.00239 (16)	0.08292 (14)	0.66351 (12)	0.0496 (4)	
H34	−0.0826	0.0852	0.6776	0.06*	
C35	0.1190 (2)	0.07876 (18)	0.71465 (13)	0.0483 (5)	
H35	0.1289	0.0776	0.7719	0.058*	
N41	0.62511 (15)	0.09036 (12)	0.57886 (9)	0.0329 (3)	
O42	0.49735 (12)	0.08624 (10)	0.57207 (8)	0.0374 (3)	
O43	0.69523 (14)	0.08635 (14)	0.65120 (8)	0.0545 (4)	
O44	0.68294 (15)	0.09930 (13)	0.51860 (8)	0.0491 (3)	
O11W	0.45059 (16)	0.26240 (10)	0.68241 (9)	0.0411 (3)	
H11X	0.389 (3)	0.303 (2)	0.6897 (16)	0.062*	
H11Y	0.499 (3)	0.311 (2)	0.6723 (16)	0.062*	
O12W	0.45109 (17)	−0.10477 (11)	0.65529 (9)	0.0453 (3)	
H12Y	0.425 (3)	−0.100 (2)	0.6035 (17)	0.068*	
H12X	0.438 (3)	−0.160 (2)	0.6658 (17)	0.068*	

### Atomic displacement parameters (Å^2^)

**Table:**

	*U*^11^	*U*^22^	*U*^33^	*U*^12^	*U*^13^	*U*^23^
La1	0.02352 (5)	0.02229 (5)	0.02468 (6)	−0.00018 (3)	0.00500 (3)	−0.00149 (3)
C11	0.0832 (18)	0.0650 (15)	0.0637 (15)	0.0058 (13)	0.0387 (14)	0.0254 (12)
C12	0.0416 (10)	0.0513 (11)	0.0344 (9)	0.0007 (8)	0.0134 (8)	0.0079 (8)
O12	0.0528 (8)	0.0354 (7)	0.0377 (7)	0.0031 (6)	0.0208 (6)	0.0043 (5)
C13	0.0549 (12)	0.0635 (14)	0.0332 (10)	0.0012 (10)	0.0214 (9)	−0.0042 (9)
C14	0.0319 (9)	0.0516 (11)	0.0362 (9)	−0.0029 (8)	0.0087 (7)	−0.0183 (8)
O14	0.0577 (9)	0.0372 (7)	0.0476 (7)	−0.0012 (6)	0.0263 (7)	−0.0107 (6)
C15	0.0545 (13)	0.0666 (15)	0.0713 (15)	−0.0042 (11)	0.0267 (12)	−0.0364 (12)
C21	0.0437 (13)	0.0679 (18)	0.153 (3)	−0.0129 (12)	−0.0071 (16)	−0.0437 (19)
C22	0.0306 (9)	0.0505 (11)	0.0451 (10)	−0.0057 (8)	0.0042 (8)	−0.0153 (8)
O22	0.0336 (7)	0.0409 (7)	0.0553 (8)	−0.0023 (5)	−0.0043 (6)	−0.0132 (6)
C23	0.0250 (8)	0.0615 (13)	0.0485 (12)	−0.0006 (8)	0.0016 (8)	−0.0030 (9)
C24	0.0326 (9)	0.0486 (11)	0.0463 (11)	0.0071 (8)	0.0069 (8)	0.0125 (9)
O24	0.0350 (7)	0.0363 (7)	0.0592 (9)	0.0022 (5)	−0.0008 (6)	0.0087 (6)
C25	0.0454 (13)	0.0614 (16)	0.143 (3)	0.0176 (12)	−0.0008 (16)	0.0247 (17)
N31	0.0256 (7)	0.0463 (9)	0.0378 (8)	−0.0009 (6)	0.0046 (6)	0.0008 (6)
C32	0.0393 (10)	0.0477 (11)	0.0365 (10)	−0.0002 (8)	0.0074 (8)	0.0017 (8)
C33	0.0360 (10)	0.0576 (13)	0.0457 (12)	0.0011 (9)	−0.0067 (9)	−0.0005 (9)
N34	0.0239 (7)	0.0671 (12)	0.0586 (11)	−0.0023 (7)	0.0093 (7)	−0.0053 (8)
C35	0.0340 (10)	0.0740 (15)	0.0381 (10)	−0.0028 (9)	0.0096 (8)	−0.0022 (9)
N41	0.0332 (7)	0.0373 (8)	0.0294 (7)	−0.0018 (6)	0.0085 (6)	−0.0029 (6)
O42	0.0294 (6)	0.0460 (7)	0.0364 (7)	0.0021 (5)	0.0048 (5)	0.0011 (5)
O43	0.0291 (7)	0.1049 (13)	0.0294 (7)	−0.0024 (7)	0.0049 (5)	0.0017 (7)
O44	0.0501 (8)	0.0691 (9)	0.0326 (7)	−0.0106 (7)	0.0198 (6)	−0.0067 (6)
O11W	0.0485 (8)	0.0256 (6)	0.0502 (8)	0.0026 (5)	0.0113 (6)	0.0023 (5)
O12W	0.0726 (10)	0.0261 (6)	0.0336 (7)	−0.0018 (6)	−0.0002 (7)	−0.0023 (5)

### Geometric parameters (Å, º)

**Table:**

La1—O14	2.4402 (12)	C22—O22	1.256 (2)
La1—O22	2.4597 (12)	C22—C23	1.392 (3)
La1—O12	2.4880 (12)	C23—C24	1.393 (3)
La1—O24	2.4939 (13)	C23—H23	0.93
La1—O12W	2.5682 (13)	C24—O24	1.261 (2)
La1—O11W	2.5808 (13)	C24—C25	1.510 (3)
La1—O43	2.6589 (14)	C25—H25A	0.96
La1—N31	2.6800 (15)	C25—H25B	0.96
La1—O42	2.7556 (13)	C25—H25C	0.96
C11—C12	1.509 (3)	N31—C35	1.312 (2)
C11—H11A	0.96	N31—C32	1.370 (3)
C11—H11B	0.96	C32—C33	1.348 (3)
C11—H11C	0.96	C32—H32	0.93
C12—O12	1.270 (2)	C33—N34	1.352 (3)
C12—C13	1.386 (3)	C33—H33	0.93
C13—C14	1.395 (3)	N34—C35	1.333 (3)
C13—H13	0.93	N34—H34	0.86
C14—O14	1.255 (2)	C35—H35	0.93
C14—C15	1.509 (3)	N41—O44	1.2311 (19)
C15—H15A	0.96	N41—O42	1.2413 (18)
C15—H15B	0.96	N41—O43	1.266 (2)
C15—H15C	0.96	O11W—H11X	0.81 (3)
C21—C22	1.508 (3)	O11W—H11Y	0.81 (3)
C21—H21A	0.96	O12W—H12Y	0.85 (3)
C21—H21B	0.96	O12W—H12X	0.73 (3)
C21—H21C	0.96		
			
O14—La1—O22	73.14 (5)	C14—C15—H15B	109.5
O14—La1—O12	69.07 (4)	H15A—C15—H15B	109.5
O22—La1—O12	108.72 (5)	C14—C15—H15C	109.5
O14—La1—O24	113.79 (5)	H15A—C15—H15C	109.5
O22—La1—O24	69.51 (5)	H15B—C15—H15C	109.5
O12—La1—O24	74.00 (5)	C22—C21—H21A	109.5
O14—La1—O12W	139.24 (5)	C22—C21—H21B	109.5
O22—La1—O12W	142.79 (5)	H21A—C21—H21B	109.5
O12—La1—O12W	78.27 (5)	C22—C21—H21C	109.5
O24—La1—O12W	78.12 (5)	H21A—C21—H21C	109.5
O14—La1—O11W	71.54 (5)	H21B—C21—H21C	109.5
O22—La1—O11W	73.17 (5)	O22—C22—C23	125.16 (17)
O12—La1—O11W	137.69 (5)	O22—C22—C21	116.06 (19)
O24—La1—O11W	138.02 (5)	C23—C22—C21	118.78 (19)
O12W—La1—O11W	126.65 (5)	C22—O22—La1	136.44 (12)
O14—La1—O43	139.68 (5)	C22—C23—C24	125.62 (18)
O22—La1—O43	71.78 (5)	C22—C23—H23	117.2
O12—La1—O43	142.12 (5)	C24—C23—H23	117.2
O24—La1—O43	70.97 (5)	O24—C24—C23	125.06 (18)
O12W—La1—O43	80.85 (5)	O24—C24—C25	116.3 (2)
O11W—La1—O43	79.79 (5)	C23—C24—C25	118.66 (19)
O14—La1—N31	74.90 (5)	C24—O24—La1	135.53 (12)
O22—La1—N31	140.50 (4)	C24—C25—H25A	109.5
O12—La1—N31	80.27 (5)	C24—C25—H25B	109.5
O24—La1—N31	146.66 (4)	H25A—C25—H25B	109.5
O12W—La1—N31	76.15 (5)	C24—C25—H25C	109.5
O11W—La1—N31	75.15 (5)	H25A—C25—H25C	109.5
O43—La1—N31	124.47 (5)	H25B—C25—H25C	109.5
O14—La1—O42	134.63 (4)	C35—N31—C32	104.87 (16)
O22—La1—O42	109.56 (4)	C35—N31—La1	127.18 (13)
O12—La1—O42	139.84 (4)	C32—N31—La1	127.95 (12)
O24—La1—O42	108.91 (4)	C33—C32—N31	109.82 (18)
O12W—La1—O42	63.95 (4)	C33—C32—H32	125.1
O11W—La1—O42	66.69 (4)	N31—C32—H32	125.1
O43—La1—O42	46.51 (4)	C32—C33—N34	106.18 (18)
N31—La1—O42	78.03 (4)	C32—C33—H33	126.9
C12—C11—H11A	109.5	N34—C33—H33	126.9
C12—C11—H11B	109.5	C35—N34—C33	107.42 (17)
H11A—C11—H11B	109.5	C35—N34—H34	126.3
C12—C11—H11C	109.5	C33—N34—H34	126.3
H11A—C11—H11C	109.5	N31—C35—N34	111.71 (19)
H11B—C11—H11C	109.5	N31—C35—H35	124.1
O12—C12—C13	124.82 (18)	N34—C35—H35	124.1
O12—C12—C11	116.26 (19)	O44—N41—O42	122.31 (15)
C13—C12—C11	118.91 (18)	O44—N41—O43	120.53 (15)
C12—O12—La1	136.74 (12)	O42—N41—O43	117.15 (14)
C12—C13—C14	125.47 (18)	N41—O42—La1	96.13 (10)
C12—C13—H13	117.3	N41—O43—La1	100.21 (10)
C14—C13—H13	117.3	La1—O11W—H11X	127.1 (18)
O14—C14—C13	124.32 (17)	La1—O11W—H11Y	135.3 (18)
O14—C14—C15	116.57 (19)	H11X—O11W—H11Y	92 (2)
C13—C14—C15	119.11 (18)	La1—O12W—H12Y	117.7 (18)
C14—O14—La1	139.35 (12)	La1—O12W—H12X	134 (2)
C14—C15—H15A	109.5	H12Y—O12W—H12X	106 (3)
			
C13—C12—O12—La1	6.4 (3)	C23—C24—O24—La1	10.7 (3)
C11—C12—O12—La1	−174.35 (15)	C25—C24—O24—La1	−168.80 (18)
O12—C12—C13—C14	−3.0 (4)	C35—N31—C32—C33	0.6 (2)
C11—C12—C13—C14	177.8 (2)	La1—N31—C32—C33	−179.32 (13)
C12—C13—C14—O14	0.9 (4)	N31—C32—C33—N34	−0.4 (2)
C12—C13—C14—C15	−179.5 (2)	C32—C33—N34—C35	0.1 (2)
C13—C14—O14—La1	−2.3 (3)	C32—N31—C35—N34	−0.6 (2)
C15—C14—O14—La1	178.08 (15)	La1—N31—C35—N34	179.36 (12)
C23—C22—O22—La1	−14.8 (3)	C33—N34—C35—N31	0.3 (3)
C21—C22—O22—La1	166.00 (19)	O44—N41—O42—La1	−179.37 (15)
O22—C22—C23—C24	−0.6 (4)	O43—N41—O42—La1	−0.25 (16)
C21—C22—C23—C24	178.6 (2)	O44—N41—O43—La1	179.40 (14)
C22—C23—C24—O24	2.3 (4)	O42—N41—O43—La1	0.26 (17)
C22—C23—C24—C25	−178.2 (2)		

### Hydrogen-bond geometry (Å, º)

**Table:**

*D*—H···*A*	*D*—H	H···*A*	*D*···*A*	*D*—H···*A*
N34—H34···O43^i^	0.86	2.15	2.942 (2)	153
O11*W*—H11*X*···O24^ii^	0.81 (3)	2.10 (3)	2.8353 (19)	152 (2)
O11*W*—H11*Y*···O12^ii^	0.81 (3)	2.00 (3)	2.8014 (19)	168 (3)
O12*W*—H12*Y*···O44^iii^	0.85 (3)	2.10 (3)	2.930 (2)	167 (3)
O12*W*—H12*X*···O22^iv^	0.73 (3)	2.30 (3)	3.0025 (19)	161 (3)

Symmetry codes: (i) *x*−1, *y*, *z*; (ii) −*x*+1, *y*+1/2, −*z*+3/2; (iii) −*x*+1, −*y*, −*z*+1; (iv) −*x*+1, *y*−1/2, −*z*+3/2.
